# Specialized Nutrition Products in Pediatric Medical Care: Clinical Applications, Formulation, Regulation, and Future Directions

**DOI:** 10.3390/nu18193298

**Published:** 2026-10-08

**Authors:** Robert D. Murray, Matthew D. Teegarden, Andie Lee Gonzalez, Mary Beth Arensberg, John T. Stutts

**Affiliations:** 1 Department of Pediatrics, The Ohio State University College of Medicine, Columbus, OH 43210, USA; murraymd@live.com; 2 Abbott Nutrition, Abbott Laboratories, Columbus, OH 43219, USA; andie.gonzalez@abbott.com (A.L.G.); john.stutts1@abbott.com (J.T.S.); 3 Nutrition Policy Insights, LLC, Columbus, OH 43016, USA

**Keywords:** medical foods, FSDU, FSMP, pediatric nutrition, medical nutrition therapy, oral nutrition supplements, enteral nutrition, pediatric malnutrition, specialized nutrition products

## Abstract

Specialized nutrition products are often used as a component of nutrition care across a spectrum of acute and chronic pediatric medical conditions. These products differ from conventional foods due to their clinical purpose, composition, and context of use, yet these distinguishing characteristics are rarely discussed collectively. The objective of this narrative review is to provide an interdisciplinary overview of specialized pediatric nutrition products, including their clinical applications, formulation considerations, regulatory environment, and future opportunities for innovation and evidence generation. These products are specially formulated and processed to provide nutrition support for vulnerable children during critical periods of growth and development. At the same time, they are subject to specialized and complex regulations. Globally, there is increased interest in the role of food formulation and processing in childhood nutrition. As policy landscapes evolve, attention should be given to specialized pediatric nutrition products to ensure continued availability and support for future research and innovations that benefit pediatric growth, development, and health outcomes.

## 1. Introduction

Nutrition is necessary for the successful growth and development of all children but takes on a critical role in children with acute or chronic conditions, growth challenges, feeding difficulties, developmental concerns, and other medical needs. Optimal nutrition status supports essential physiological processes including metabolism, immunity, tissue repair, and inflammatory responses, which are central to health maintenance and recovery from critical illness [1,2]. However, prolonged illness or complex conditions themselves increase malnutrition risk through various mechanisms including inadequate intake, impaired nutrient absorption, increased metabolic requirements, dietary restrictions, and other challenges [3]. Careful management of these risk factors is necessary to prevent and treat malnutrition and support growth and development for the pediatric population with special needs.

The estimated prevalence of malnutrition in the form of undernutrition ranges from 30% to over 50% among critically ill children [4,5] and children with chronic diseases [6,7], depending on care setting and socio-demographic factors. The Academy of Nutrition and Dietetics and the American Society for Parenteral and Enteral Nutrition (ASPEN) have defined pediatric malnutrition as “an imbalance between nutrient requirements and intake that results in cumulative deficits of energy, protein or micronutrients that may negatively affect growth, development and other relevant outcomes” [8]. Left untreated, pediatric malnutrition during an illness has been associated with higher mortality risk, need for mechanical ventilation and longer duration thereof, and longer pediatric intensive care unit and hospital lengths of stay [9]. Adverse outcomes such as developmental delay, intellectual deficits, and increased care costs may result [6]. Nutrition interventions for malnutrition are thus a critical component of care.

For many children with acute and chronic conditions, usual dietary intake is often insufficient to meet their nutrition needs. Additional nutrition intervention may be required to address malnutrition and promote growth and development [10]. The products used to provide this support, including oral nutrition supplements (ONSs), enteral formulas, and medical foods, among others, are defined in Table 1. Collectively these products are often referred to as specialized (pediatric) nutrition products.

Globally, there is increased interest in childhood nutrition and the role of food formulation and processing [14]. Policy efforts in this area tend to focus on the general pediatric population, yet unintended consequences that impact access to specialized pediatric nutrition products could occur if policies do not consider the science and intention behind these specialized products. The need for balancing clinical indication with broader public health policy efforts has been recently reviewed by JCY Louie [15]. Part of the challenge is a lack of awareness that these products are fundamentally different from conventional foods. They are intentionally formulated and processed to meet the very specific nutrition needs of children with acute and chronic conditions, and their ingredients are carefully selected to serve these needs. A comprehensive understanding of what specialized pediatric nutrition products are and how they are used is critical for assuring access to these products that help sustain vulnerable children and address malnutrition risk.

The objective of this narrative review is to help support this understanding by discussing multiple dimensions of specialized nutrition products that are rarely described together in existing literature, especially with a focus on pediatric populations. Specifically, the review discusses interdisciplinary aspects of these products, including their clinical application, formulation, and global regulatory environment. Also presented are an illustrative hypothetical case study and description of opportunities for future product research and advancement. As regulatory and policy landscapes further evolve, attention to this special group of products is required to ensure their continued availability and to support future innovations.

## 2. Narrative Review Scope

This narrative review is intended to provide a high-level overview and synthesis of interdisciplinary aspects related to specialized pediatric nutrition products. Relevant literature and clinical guidelines published in the past five years were prioritized when available. All study types were considered. This review is not intended to provide a comprehensive overview or detailed list of each clinical condition where specialized pediatric nutrition products may be used as an element of nutrition care, nor does it evaluate the weight of evidence regarding specialized nutrition product use in each of these conditions. Furthermore, products for children under one year of age (infant formula), for resource-related starvation (ready-to-use therapeutic food), and parenteral (intravenous) nutrition are outside the scope of this review.

Malnutrition can occur in several forms ranging from undernutrition (underweight, wasting, stunting) to overnutrition (overweight, obesity) [16]. Acute pediatric malnutrition has been defined as illness-related and occurring for less than three months, whereas chronic malnutrition lasts for more than three months and is not illness-related (it is behavioral or socioeconomic-related) [8]. In this review, the term malnutrition refers to the undernutrition form related to illness, medical condition, and/or behavior. The scope of the clinical portion of this review is limited to those conditions with a higher incidence of occurrence and medical nutrition need in the United States (US) pediatric population. For more in-depth information on nutrition interventions for each of these conditions, readers are directed to the literature cited in this review and authoritative publications from organizations such as the American Academy of Pediatrics.

## 3. Clinical Role and Impact

Pediatric patients with complex health conditions may be at an increased risk for malnutrition due to a reduction in dietary intake, but other risk factors such as malabsorption, increased nutrient loss, altered nutrient requirements, and increased energy expenditure can also contribute to the malnutrition risk [17]. Because of this, care may involve multidisciplinary teams of healthcare professionals focused on optimizing the patient’s nutrition. Specialized pediatric nutrition products are common interventions to address particular nutrition needs. This section briefly discusses key conditions in which nutrition management plays a specialized role in pediatric patient care and identifies how different product types may contribute to positive patient outcomes. Table 2 summarizes these conditions, their primary mechanisms of nutrition risk, and potential interventions. While these products may provide critical intervention during certain acute periods and in ongoing medical care, routine assessment and close monitoring are necessary to determine when and how nutrition care plans should be adjusted for a growing child’s changing nutrition and clinical needs.

Nutrition-related pediatric conditions vary widely in prevalence. Among the most prevalent are faltering weight (5–10%) [18], food allergies (~7–8%) [19], and developmental disorders (~8.5%) [20]. Other conditions often require specialized nutrition care, such as pediatric oncology, gastrointestinal disorders, cystic fibrosis, metabolic disease, severe trauma, and renal disease, as well as complex surgical conditions. These conditions consistently disrupt appetite, nutrient intake, absorption, and/or metabolic demand, placing children at increased risk for malnutrition (including hospital-acquired malnutrition), poor growth, delayed neurodevelopment, and impaired immunity with a negative impact on quality of life and health outcomes as described in further detail below.

### 3.1. Faltering Weight (FW)

Weight gain is fundamental to a child’s growth and development. Unexpected weight loss or a change in weight gain from a child’s typical growth pattern are primary signs of malnutrition risk and can occur across a number of pediatric conditions. Failure to thrive, now known as faltering weight (FW), refers to inadequate weight gain or growth in a child [21]. It is a multifactorial condition often rooted in insufficient caloric intake relative to needs due to a variety of organic (e.g., chronic illness or medical condition) or non-organic (e.g., improper feeding practices or environmental/social factors) causes. If untreated, prolonged malnutrition in FW can lead to short stature, developmental delays and other long-term cognitive and/or health consequences [18]. The primary goal of nutrition care is to provide sufficient energy and nutrients to promote catch-up growth, while evaluating and addressing the underlying cause of poor intake. Interventions will differ based on the individual patient and underlying causes of faltering weight. A hypothetical case study involving FW with mild malnutrition is summarized in Box 1.

Box 1 Hypothetical Pediatric Case Study Example: Faltering weight with mild malnutrition. ^a^ Clinical Classification Criteria: A child is considered to have faltering weight if any one of the following is present: weight-for-length OR BMI **-for-age < −1.65 z-score (~<5th percentile), weight gain velocity < −2 z-score * for age, or decline in weight, weight-for-length, or BMI ** ≥ 1 z-score * over time [21]. ^b^ Pediatric malnutrition is defined as an imbalance between nutrient requirements and intake that results in cumulative deficits of energy, protein, or micronutrients that may negatively affect growth, development, and other relevant outcomes [8]. ^c^ Pediatric nutrition-focused physical exam classifications (subcutaneous fat loss, muscle wasting) [22]. ^d^ Pediatric malnutrition indicators and diagnosis [23]. * z-score is a calculation used to determine how far a child’s growth measurement (such as weight, length/height, BMI) deviates from the population mean based on a child’s age and sex. ** BMI: Body mass index is an estimation of body fat based on height and weight measurements.

Pediatric Condition

Faltering weight ^a^ (formerly failure to thrive) in a toddler

Pediatric Primary Mechanisms of Nutrition Risk ^b^

- Inadequate intake for the last month (feeding disorder, food selectivity)
- Elevated nutrient requirements (rapid growth period)

Nutrition-Focused Physical (NFPE) Exam Findings ^c^

- Limited subcutaneous fat at triceps and orbital regions
- Poor calf muscle tone and bulk
- No edema; normal skin turgor
- Fatigue during meals, limited endurance for textured foods

Malnutrition Classification ^d^

Consistent with mild acute (<3 months) pediatric malnutrition when NFPE findings are interpreted alongside declining weight-for-length z-score *

Pediatric Nutrition Support Strategy ^a^

- Age-appropriate pediatric oral nutrition supplement (polymeric)
- Energy-dense formulation (≈1.0–1.5 kcal/mL) to limit volume displacement
- Used to augment, not replace, family foods

Timing, Monitoring, and Evaluation

- Administer between or after meals (1–2 servings/day)
- Monitor weight and weight-for-length *z*-score every 2–4 weeks
- Reassess NFPE fat and muscle stores over time
- Adjust volume or caloric density if growth trajectory does not improve within 4–6 weeks

Addressing FW often requires a collaborative, and potentially long-term, approach in which pediatricians, dietitians, and sometimes behavioral specialists work with the family to improve feeding practices and the caloric density of the child’s diet [18,24]. Initial steps often focus on educating caregivers about high-calorie, nutrient-dense foods, which can include nutrition products such as ONSs. If outpatient measures fail or the child is severely malnourished, more intensive intervention, including feeding via a nasogastric/gastrostomy tube, may be initiated. Updated clinical practice guidelines for FW were recently published by the American Academy of Pediatrics (AAP) and the North American Society for Pediatric Gastroenterology, Hepatology and Nutrition (NASPGHAN) and are summarized in Box 2. Since FW is an early indicator of pediatric malnutrition risk, these clinical recommendations support timely identification, evaluation, and nutrition intervention.

Box 2 Summary of American Academy of Pediatrics (AAP) and North American Society for Pediatric Gastroenterology, Hepatology and Nutrition (NASPGHAN) clinical practice guidelines for faltering weight (FW). Reference: Adapted from [21]. * BMI: Body mass index is an estimation of body fat based on height and weight measurements. ** *z*-score is a calculation used to determine how far a child’s growth measurement (such as weight, length/height, BMI) deviates from the population mean based on a child’s age and sex.

1. The definition of faltering weight includes any of the following:
  - Weight-for-length, or BMI *-for-age < −1.65 *z*-score ** (5th percentile);
  - In children aged < 2 years, weight gain velocity < −2 *z*-score ** for age (2.3rd percentile);
  - Decline in weight, weight-for-length, or BMI * ≥ 1 *z*-score **.
2. Accurate anthropometric measurements are essential to monitor growth both in all children and in the diagnosis of faltering weight.
3. The FW Guideline Panel does not recommend endoscopy in the initial workup of children with faltering weight. Endoscopy with biopsy may be considered, however, in children who have persistent faltering weight or for whom there are concerns for conditions that cannot be diagnosed without endoscopy.
4. In children without specific signs, symptoms, and findings that would prompt a focal evaluation, the FW Guideline Panel recommends against diagnostic testing as part of the initial routine workup for faltering weight.
5. The FW Guideline Panel recommends against using socioeconomic status as an assigned risk factor when diagnosing faltering weight.
6. In children who have faltering weight, the FW Guideline Panel recommends the use of increased calories in the form of oral nutrition supplementation.
7. In children who have faltering weight and documented feeding issues, the FW Guideline Panel recommends therapy for pediatric feeding disorder.

### 3.2. Severe Food Allergies and Allergic Reactions

Studies show that children with food allergies tend to have slower growth in both height and weight compared to their peers without food allergies [25]. Undernutrition and FW are also frequently reported for children with eosinophilic esophagitis [26], a chronic, immune-mediated inflammatory disease of the esophagus, primarily driven by allergic reactions. Children with multiple or severe allergies and children with eosinophilic esophagitis often have very restricted diets (to eliminate allergens), increasing their risk of nutrient deficiencies [27,28]. Nutrition management for food-allergic children focuses on finding safe, nutritionally equivalent substitutes for the eliminated foods [25,27]. For example, a child with severe or multiple food allergies may require an elemental formula to ensure adequate protein and energy intake.

### 3.3. Neurological, Behavioral, and Developmental Disorders

Children with neurological impairments or developmental disorders are at high risk of experiencing nutrition problems, such as undernutrition, FW, and micronutrient deficiencies, predominantly due to feeding difficulties [29,30]. For example, children with cerebral palsy experience motor disability, often accompanied by oral motor dysfunction, and have a greater risk of malnutrition [31]. Many patients may have problems with chewing, swallowing, or self-feeding which can impact mealtimes, increase aspiration risk, and lead to an overall inadequate intake of calories [31,32]; an estimated 30–46% are at risk for malnutrition [33]. Nutrition intervention revolves around adapting feeding methods and maximizing caloric density based on individual needs [34]. Interventions include modifying food texture (purees, thickened liquids), using specialized feeding methods, and potentially supplementing with a high-calorie ONS.

Autism spectrum disorder (ASD) is a heterogeneous and complex developmental and neurological condition. Nutrition as an environmental factor affecting ASD, as well as a factor in helping alleviate some of the behavioral and gastrointestinal symptoms of the condition, is an active area of research [35]. While most children with ASD are not severely growth-impaired, they are often nutritionally vulnerable due to selective or restrictive eating behaviors [36] that can include a very limited repertoire of foods [37]. Despite sufficient total calories, low protein, iron, and/or vitamin intakes are not uncommon and can lead to FW and/or nutrient deficiencies [38,39,40]. Nutrition management focuses on behavioral feeding interventions and supplementation to ensure adequate nutrition intake. If children with ASD are unable to take adequate food or ONSs orally, tube feeding may be considered [41]. Gastrointestinal (GI) problems (including constipation, diarrhea, and abdominal pain) are additional common comorbidities in autism spectrum disorder [42] which can also be addressed through diet, such as through the use of a high-fiber ONS.

### 3.4. Cancer

Children with cancer have significant nutrition challenges and risk for malnutrition [43]. Many pediatric cancers (such as leukemias, lymphomas, or solid tumors) create a state of increased energy expenditure and protein catabolism. Additionally, cancer therapies frequently induce anorexia, nausea, vomiting, mucositis, and taste changes, all of which may drastically reduce oral nutrition intake [44]. Children undergoing treatment may experience slowed or ceased linear growth and nutrition deficiencies that can lead to loss of adipose tissue, lean tissue, and essential nutrients necessary for recovery and overall health [44,45]. Interventions can range from dietary counseling and high-calorie/high-protein meal plans to the use of specialized nutrition products to increase nutrient intake [46]. ONSs are indicated when a child is at moderate risk of malnutrition or unable to meet daily requirements through regular food alone [46], and recently reviewed evidence suggests that ONSs may improve weight status across various malignancy types [47]. Tube feeding is suggested when there is a functioning gut, but daily intake requirements are unable to be met orally [46]. Without use of specialized pediatric nutrition products during therapy, support of immune function and healing could be hindered in these patients [46,48].

### 3.5. Gastrointestinal Disorders (Crohn’s Disease, Short Bowel Syndrome)

Chronic GI diseases in children often lead to malnutrition through a combination of mechanisms that include malabsorption, increased nutrient losses, and reduced intake. Crohn’s disease, an inflammatory bowel disease, exemplifies this risk [49]. Children with Crohn’s disease often experience weight loss or FW due to reduced dietary intake (from pain, nausea, or anorexia), loss of proteins because of intestinal mucosal inflammation, and/or malabsorption [50,51]. This can lead to micronutrient deficiencies and delayed puberty or stunting if not addressed [50]. Pediatric Crohn’s disease commonly presents during periods of rapid growth, so the impact on development can be significant if nutrition status is not optimized [24]. Because many children with Crohn’s disease require nutrition supplementation with the goal of optimizing nutrient absorption [52], nutrition has been recommended as a first-line therapy to help induce remission. Nutrition intervention involves providing a high-protein/high-calorie diet, often including ONSs to promote catch-up growth and micronutrient supplementation to address specific deficiencies, such as iron for anemia [53] or vitamin D for bone health [54]. Exclusive tube feeding may be used as a strategy to induce remission while also improving nutrition status [55,56].

Short bowel syndrome represents another GI condition, which can occur when a portion of the intestine has been resected or is non-functional, leading to insufficient absorptive surface area. This condition can cause profound malnutrition and poor growth [57]. Children with short bowel syndrome cannot fully absorb fluids and nutrients from food, leading to chronic diarrhea, malabsorption, and electrolyte imbalances [58]. In addition, some children can have feeding challenges related to rapid intestinal transit and gastric acid hypersecretion, which can further impair nutrient uptake [57]. Management requires highly specialized, multidisciplinary nutrition intervention and a comprehensive approach that typically includes a combination of enteral tube feeding and parenteral nutrition (IV feeding) as the remaining GI mucosa adapts and recovers [59,60].

### 3.6. Pulmonary Disease (Cystic Fibrosis)

Cystic fibrosis is a genetic disorder characterized by viscid secretions affecting the respiratory and digestive systems [61]. Children with cystic fibrosis are medically complex, with chronic lung infections, exocrine pancreatic insufficiency, dysfunction of digestive organs, and impaired digestion. Malnutrition remains a significant concern in pediatric cystic fibrosis. Patients are prone to increased metabolic demands and malabsorption of nutrients. which may prevent adequate nutrition intake needed to support growth, pulmonary health, and overall clinical outcomes [61,62]. Nutrition intervention typically includes optimizing calorie, fat, and fat-soluble vitamin intake paired with pancreatic enzyme replacement therapy. Specialized ONSs such as those made with peptide-based protein systems to promote easier protein digestion and/or specialized lipids to enhance fat absorption, may be integrated into cystic fibrosis management if growth and nutrient status cannot be maintained by regular foods alone. Tube feeding may be necessary in cases where oral intake is insufficient [63].

### 3.7. Inherited (or Inborn) Metabolic Diseases

Inborn errors of metabolism are genetic disorders, such as Phenylketonuria (PKU), Maple Syrup Urine Disease (MSUD), urea cycle disorders, and many others, that disrupt the body’s normal processing and absorption of key nutrients [64]. These conditions often use targeted medical foods as a cornerstone of treatment [65]. For example, patients with PKU require specially formulated phenylalanine-free medical foods to provide other amino acids [66], while those with MSUD require medical foods that are branched-chain amino acid-free [67]. Often fortified with micronutrients, these medical foods allow patients with inborn errors of metabolism to receive adequate nutrition and to increase amino acid intake while navigating complicated, restrictive diets. Other therapies for inborn errors of metabolism, such as the pharmaceutical sapropterin for PKU [68], may enable dietary liberalization and reduced dependence on protein substitutes or specialized medical foods. Even still, continued nutrition monitoring and support remain important components of long-term care.

### 3.8. Trauma, Burns, and Surgery

Severe injuries, major burns, and extensive surgical interventions can precipitate a state of hypermetabolism and hypercatabolism in children, placing them at nutritional risk [69]. Prolonged hypermetabolism from burns or critical illness has been directly associated with stagnant linear growth [70]. Children recovering from major surgeries (such as extensive gut resection or cardiac surgery) or multi-trauma may be in a sustained catabolic state and lose lean body mass essential for growth and development. The objectives of nutrition intervention are to manage hypermetabolic responses and provide sufficient protein and calories to meet the vastly elevated needs for wound healing and catch-up growth [69]. Nutrition products provided orally or by tube may be necessary to support sufficient nutrient intake [71,72,73]. Continuous monitoring and adjustments are essential, since the metabolic rates of these patients may change as they recover [74].

For children who require surgery and are malnourished, optimizing nutrition intake prior to surgery is recommended. Nutrition interventions include oral or tube-fed products [72]. In addition, recent multidisciplinary guidelines highlight the value of early postoperative tube feeding to help maintain gut function [73].

### 3.9. Renal Disease

Renal disease in children is accompanied by a cascade of nutrition and growth challenges [75]. Children with renal disease, specifically chronic kidney disease, often experience malnutrition and impaired growth due to a combination of factors like protein energy wasting and micronutrient deficiency [76]. Poor appetite can result from delayed gastric emptying, gut dysmotility, and reduced sense of smell and taste [77]. In addition, dialysis can contribute to nutrient losses and risk for growth impairment [75]. Sufficient energy intake is often considered essential for these patients. However, micronutrient needs are also critical. For example, active vitamin D, calcium, and phosphate binders are used to counteract renal bone disease; iron and erythropoietin address anemia that can otherwise stunt appetite and growth. Conversely, nonessential elements such as aluminum can be harmful in excess [78]. In pediatric renal disease, nutrition risk may arise also from the need to restrict protein, potassium, phosphorus, sodium, and fluids, combined with nutrient losses during dialysis that can increase protein and micronutrient requirements [77]. Specialized pediatric nutrition products, such as ONSs, therefore become vital tools in managing the multifaceted nutrition needs of renal patient care [77].

## 4. Formulation Considerations

As described above, a range of pediatric health conditions can impact a child’s nutrition status, increase their risk for malnutrition, and shape their need for specialized nutrition products. Similar to those intended for adult populations, there are key factors that must be considered when designing formulations for children. Understanding these products from a food science perspective can be helpful in optimizing their development, use, and access.

For specialized nutrition products to be effective, they must provide the correct balance of nutrients for the intended population and maintain these nutrient values over an extended product shelf life. These products must also exist in forms that are compatible with the intended feeding mode and ultimately be consumed by the patients for whom the products are recommended. Compared to conventional foods, the amount and variety of nutrients in specialized nutrition products impart unique challenges that must be overcome in product design. These products are formulated using a wide range of ingredients, including intact proteins, peptides, free amino acids, various oils, vitamins, mineral salts, and other functional components [79]. Importantly, every ingredient serves a necessary function either for nutrition, formulation stability and quality, and/or organoleptic acceptance. Some ingredients can serve multiple functions. Carbohydrates, for example, are an important macronutrient but can also have a role in the taste, flavor, and the physical matrix of a product [80].

Specialized nutrition products are typically processed using a “wet-batch” approach. Briefly, ingredients are combined into liquid formulations that are subjected to several mixing and heat treatment steps. These mixtures are then filled into containers and commercially sterilized, aseptically filled into containers, or spray-dried into powdered products. All three processes expose products to additional heat [81]. Although heat treatment is necessary for processing and food safety considerations, it can introduce technical challenges that must also be addressed.

Exposure to heat and oxygen are important drivers of nutrient instability during processing, but various aspects of product formulation may also modulate losses. For example, product form (liquid vs. powder), product pH, the presence of metal ions, and the degree of protein hydrolysis can all influence nutrient stability [79,82]. These factors can also impact nutrient loss during product storage, and in many cases, labile nutrient degradation is a key factor determining product shelf life [79]. Products are often fortified at levels that account for nutrient degradation, but other ingredients, such as emulsifiers and antioxidants, can help improve nutrient stability too [83]. Nutrient levels can be further maintained over shelf life by optimizing packaging materials and flushing package headspace with inert gases [79,81]. Since a variety of factors influence nutrient stability, each formulation will have unique considerations to ensure nutrient label claims are achieved throughout shelf life.

Beyond nutrient stability, the design of specialized nutrition products must account for other characteristics that are important for their intended functions. For instance, product osmolality can affect patient tolerance of a formula, especially in patients with gastrointestinal diseases [82]. Nutrient bioavailability is another factor that is important to consider. The forms, levels, and overall blend of nutrients, as well as composition of the final product matrix, can affect nutrient absorption once the product is consumed [84]. This is especially important when products are intended for use in patients with malabsorption disorders. A simple example of this is the use of medium chain triglyceride oil as a fat source in ONSs for patients with fat malabsorption. Viscosity can also impact product functionality, especially when products are intended for tube feeding. Tube feeding products must be formulated so that they can flow easily and safely at a set rate through a narrow tube without clogging [85].

The sensory properties of foods, including taste, aroma, and texture, are key drivers of their acceptance, which can translate to overall patient adherence to healthcare professional recommendations of nutrition products [86]. Certain ingredients such as amino acids, and the overall concentrated levels of nutrients within these products, can impart negative, often bitter, tastes to formulations [87]. This poses a particular challenge when designing nutrition products for pediatric populations, as children tend to have a pronounced, innate aversion to bitter flavors [88]. Additionally, children undergoing certain treatments, like chemotherapy, may have an altered sense of taste [89]. Children’s sensory preferences may also change over time. In particular, texture may influence the liking of a food more in early childhood, while the flavor of a food plays a larger role as children mature [90]. Thus, flavors are necessary ingredients in specialized pediatric nutrition products, as they mask undesirable sensory qualities and help promote patient compliance when these products are recommended [80].

## 5. Regulatory Considerations

Regulations heavily influence how specialized pediatric nutrition products are formulated, processed, labeled, and placed on the market, and the global regulatory environment governing these products is complex. At a high level, these products are regulated as foods but are recognized as specialized foods intended to fulfill specific needs that are distinct from those of the general, healthy population. This section focuses on applicable frameworks for pediatric specialized nutrition products, including Codex Alimentarius (Codex) standards and the regulations in the US, European Union (EU), and Canada.

Codex is a collection of global food standards jointly established by the World Health Organization and the Food and Agriculture Organization of the United Nations. The Codex Commission consists of 189 member countries and the EU, and Codex standards, guidelines, and codes of practice have a widespread impact on the global food regulatory environment. Within Codex, the specialized nutrition products considered in this review fall under the standards for foods for special dietary use (FSDU; CXS 146-1985) and the subcategory of foods for special medical purposes (FSMP; CXS 180-1991). These two general standards provide regulatory definitions and principles for indications for use, labeling, and claims [91]. Local regulatory bodies may also build upon Codex standards with additional criteria, including labeling and composition requirements.

**Figure 1:**
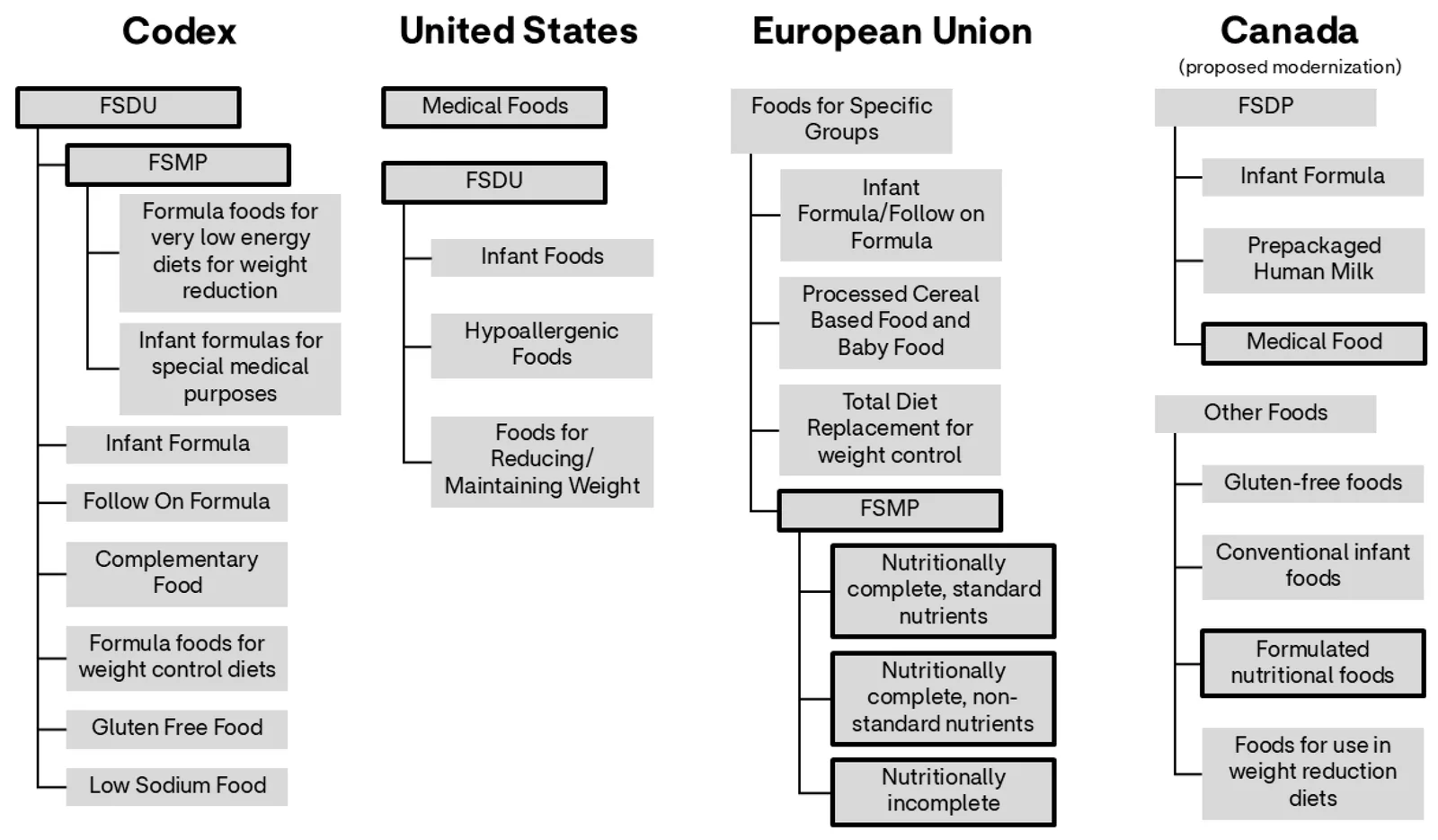
Regulatory classification of specialized pediatric nutrition products across four regulatory authorities. Bolded boxes indicate specific categories relevant to specialized pediatric nutrition products as defined in this paper. Abbreviations: Food for special dietary use (FSDU); food for special medical purpose (FSMP); food for special dietary purpose (FSDP).

While many countries largely reference or adopt Codex for food regulations that pertain to specialized nutrition products, others like the US, EU, and Canada have more developed local frameworks instead. In the US, these products may be categorized as FSDU (21 CFR 105.3) or medical foods (21 CFR 101.9(j)(8)), while in the EU they fall under a single FSMP category (Regulation No 609/2013) with three defined subcategories (Commission Delegated Regulation 2016/128). The similarities and nuanced differences between different frameworks are important to understand, as they can influence product innovation and accessibility. For example, experts across academia, industry and government in Canada recently concluded that the current regulatory framework in their country limited the population’s access to specialized nutrition products and beneficial product innovations available in other countries [92]. In response, Health Canada recently published a proposed modernized framework aimed at clarifying product categories, updating science-based guidelines, and encouraging innovation [93]. Under this draft framework, specialized nutrition products may be categorized in Canada as either formulated nutritional foods or medical foods. Codex, US, EU, and Canadian regulatory frameworks are summarized in Figure 1.

Although relevant categories may differ slightly across regulatory bodies, they can be grouped into analogous categories based on their intended use: medical foods or FSMP which are intended to be used under medical supervision and FSDU or fortified nutritional foods (often referred to as ONSs in the scientific literature) that are generally available and able to be self-selected by individuals who need to meet a special dietary need. It is important to note that a healthcare practitioner may still recommend use of an FSDU or fortified nutritional food. The delineation between these general groupings of product categories, their regulatory definitions, and the importance of localized interpretation of regulations has been extensively discussed elsewhere [92,94]. In the US, FSDU can be thought of as products that promote the day-to-day health of individuals living with certain conditions versus medical foods that are core components of disease management [94].

Across regulatory bodies, specialized pediatric nutrition products are defined in ways that, either explicitly or by regulatory context (e.g., unique nutrient composition requirements or notification requirements), distinguish them from conventional foods. The concept of “specially formulated and processed” appears across jurisdictions, as does the idea that these special foods support dietary management that is not possible with conventional foods alone. Regardless of specific terminology, global regulations clearly indicate that products within these categories must have a health and/or medical basis. Further distinguishing these products from conventional foods, specialized nutrition products may be subject to specific composition, labeling, and registration requirements and may have unique exemptions depending on their categorization. In the US, for example, medical foods are exempt from standard nutrition labeling to allow for more tailored labeling needs. Expertise in applicable regulations for each global market is essential to ensure compliant product formulation, manufacture, and availability.

## 6. Areas for Future Research and Advancement

While specialized nutrition products have been shown to improve outcomes for vulnerable pediatric populations, gaps remain in long-term outcomes research and technology integration, health policy and quality metrics, and product innovation and study design. While some advances in these areas have been made for adult populations, pediatric-specific evidence remains limited.

### 6.1. Long-Term Research and Technology Integration

Evidence supporting specialized pediatric nutrition products has traditionally been focused on short-term pediatric outcomes like weight gain [95], with improved weight gain demonstrated in some cases as soon as 10 days [96]. More research is needed to examine long-term pediatric impacts in areas such as rehabilitation and recovery [97], including when nutrition is combined with exercise to preserve muscle mass [98], the microbiome [99], and neurodevelopment [100].

Opportunities also exist for research on digital health integration. For example, remote monitoring of nutrition product intake and tolerance is a nascent area of research despite its potential to improve adherence and outcomes [101]. There is evidence that interventions such as pediatric enteral nutrition after hospital discharge can benefit from telemedicine when combined with a multidisciplinary follow-up plan [102], but the integration of telemedicine with digital platforms for monitoring product intake, growth metrics, and tolerance could enhance personalized care and provide real-time feedback to parents and caregivers [103,104].

Artificial intelligence (AI)-driven tools could support innovation too. While not specific to pediatrics, future AI nutrition advancements have been discussed that could assist clinicians in early evaluation and determining nutrition requirements, predicting complications, and optimizing nutrition interventions [105,106]. However, cautions have been raised about using AI-based dietary recommendations without professional supervision, as current algorithms can be prone to error in nutrient calculations [107]. Though evidence of long-term clinical effectiveness in specific pediatric populations is still limited [108], emerging frameworks may facilitate research in this area [109].

Continued research on long-term outcomes of specialized pediatric nutrition products and validation of emerging technologies in pediatric populations may provide meaningful improvements in patient care.

### 6.2. Health Policy and Quality Metrics

In the US, there is an increased awareness of and interest in the role of nutrition in preventive care, which has contributed to the growth of “food as medicine” [110] and the federal government’s Make America Healthy Again [111,112] health policy initiatives. Yet, policy frameworks governing access and reimbursement for specialized nutrition products remain fragmented. US coverage for these products often varies by state, insurer, and product type, which can create challenges in access for medically warranted nutrition intervention [113]. Globally, coverage for these products may also vary depending on country, region, and healthcare system.

Quality metrics can be important in helping support nutrition product access and reimbursement. For example, a quality metric related to the nutrition care process and potential use of nutrition products for adult malnutrition, specifically the US Malnutrition Care Score (formerly the Global Malnutrition Composite Score) [114], has advanced to benefit improved health outcomes [115]. However, pediatric-specific malnutrition care metrics and quality improvement programs remain underdeveloped [116], even as there have been efforts to promote the Malnutrition Care Score for pediatric populations [117]. This has limited both health system accountability and quality improvement efforts to address malnutrition in pediatric hospital and outpatient care [116].

### 6.3. Product Innovation and Study Design

While there are a range of specialized pediatric nutrition products available today, current formulations may not fully meet the many nuanced needs of the pediatric population and thus there are multiple opportunities for product innovation. For example, products administered orally must be palatable [118]. Taste aversions can be a concern for maintaining ONS use [119]. While different flavoring agents and other components can be used to help mask odor, texture, and taste [120], future advancements could leverage novel delivery systems such as nanoencapsulation of specific nutrients or other ingredients. Nanoencapsulation could help mask the strong tastes of certain components and enhance nutrient delivery, stability, and gastrointestinal tolerance [121,122].

There are also opportunities for further optimizing product formulations to meet the complex nutrition needs of certain pediatric diseases and conditions such as congenital heart disease [123], Crohn’s disease [124], neurological disorders [125], and cystic fibrosis [63]. Emerging evidence additionally highlights the influence of the gut microbiome on digestion, immunity, and neurodevelopment. Research is needed to further understand how prebiotics, probiotics, and potentially other components [126] could be included in specialized nutrition products to improve microbial balance and promote positive health outcomes in the context of pediatric diseases.

Nutrition research study design is an opportunity for product innovation as well. While randomized controlled trials (RCTs) remain the gold standard in nutrition research, the complexity of clinical conditions, nutrition interventions, and other factors can be difficult to fully control. Alternative study designs are being explored as potential approaches to advance nutrition research and care standards, including pragmatic (real-world evidence) trials and those with adaptive designs that allow for pre-planned trial modifications [127].

Addressing these opportunities and gaps requires a multi-pronged approach: investing in rigorous research on long-term outcomes, advancing technology and AI integration, harmonizing policy goals with coverage availability and quality metrics, and bringing innovations in product development and research design to the forefront. Collaboration among clinicians, policymakers, and industry will be essential to ensure that specialized pediatric nutrition products continue to be advanced and remain safe, effective, and accessible for all children who need them.

## 7. Limitations

This narrative review offers a high-level interdisciplinary perspective on key factors pertaining to specialized pediatric nutrition products. Readers should not interpret this as a systematic review because the literature selection was not based on a predefined and reproducible search strategy, so relevant studies may have been unintentionally omitted. Further, since the review is presented at a high level, the evidence discussed across pediatric conditions is heterogeneous with respect to several factors, which may limit direct comparisons and broad generalization. Similarly, as noted in this review, there are substantial and nuanced differences across national and regional regulatory bodies that may limit the international applicability of some conclusions.

## 8. Conclusions

Specialized pediatric nutrition products can play critical roles in supporting nutrition, growth, development, and dietary management of certain diseases in children with acute and chronic medical conditions. Their use can help support individualized nutrition care when used in concert with clinical evaluation, monitoring, and periodic reassessment. These products are intentionally formulated and processed to satisfy specialized nutrition needs and are governed by complex regulations that can differ across countries and regions. Because of their unique nature and use, special consideration should be given for specialized pediatric nutrition products in nutrition policies. Further research is needed to understand long-term outcomes of specialized nutrition product use in pediatric populations, and opportunities exist for the integration of emerging technologies in product formulation and administration. Healthcare providers, nutrition professionals, food scientists, regulatory and health policy authorities, and elected officials all play pivotal roles in maintaining access to and further advancement of these products to meet specific child nutrition needs and thus to support positive pediatric health outcomes.

## Figures and Tables

**Table 1:** Definitions of specialized nutrition product terms used in patient care.

Term	Definition	Reference
Oral nutrition supplement	General term for commercial products that provide nutrition support for patients and complement oral dietary intake of normal foods or provide sole source oral nutrition; these products can sometimes also be fed by tube.	[11]
Enteral nutrition/enteral formula	Formulations fed directly into the gastrointestinal tract (e.g., via feeding tube).	[12]
Polymeric formulas	Products made with fully intact macronutrients	[12,13]
Oligomeric/monomeric/elemental formulas	Products made with macronutrients that have been enzymatically hydrolyzed into, or completely formulated using, elemental nutrient components (e.g., contain amino acids vs. whole proteins).	[12,13]
Medical food (United States legal definition)	“The term “medical food” means a food which is formulated to be consumed or administered enterally under the supervision of a physician and which is intended for the specific dietary management of a disease or condition for which distinctive nutritional requirements, based on recognized scientific principles, are established by a medical evaluation.”	Section 5(b) of the United States Orphan Drug Act ^1^

^1^ 21 U.S. Code § 360ee(b)(3)).

**Table 2:** Common pediatric conditions, their primary ^1^ mechanisms of nutrition risk, and potential nutrition interventions.

Pediatric Condition	Inadequate Intake	Malabsorption of Nutrients	Elevated Nutrient Requirements	Dietary Restrictions	Potential Oral/Enteral Nutrition Intervention and Specialized Nutrition Products ^3^
Faltering Weight	x	x	x	x	Calorie fortification, oral nutrition supplement, enteral feeding with high-calorie polymeric formulas
Severe Food Allergies	x			x	Elimination diet management using extensively hydrolyzed formula (EHF), amino acid formula (AAF)
Neurological, Behavioral and Developmental Disorders	x			x ^2^	Oral supplementation; enteral feeding using polymeric pediatric formulas
Pediatric Oncology	x		x		Early nutrition intervention; enteral nutrition using polymeric or peptide formulas
Gastrointestinal Disorders (Crohn’s Disease, Short Bowel Syndrome)	x	x			Exclusive enteral nutrition using polymeric formulas (Crohn’s disease); intestinal rehabilitation using peptide/semi-elemental/elemental formulas (short bowel syndrome)
Pulmonary Disease (Cystic Fibrosis)	x	x	x		High-calorie/high-protein therapy using high-calorie polymeric formulas
Metabolic Disease	x	x		x	Disease-specific dietary therapy using medical foods
Severe Burns or Major Trauma	x		x		Early enteral feeding using high-protein formulas
Renal Disease	x	x	x	x	Protein/electrolyte management using renal-specific formulas

^1^ Primary mechanisms are denoted, but this does not exclude the possibility that a condition may involve other risk mechanisms. ^2^ Self-limited diet in autism spectrum disorder. ^3^ Potential interventions shown are representative examples and not an exhaustive list of treatment recommendations. Clinical use should be guided by individual patient needs, product availability, provider expertise, and applicable clinical practice guidelines.

## Data Availability

No new data were created or analyzed in this study. Data sharing is not applicable to this article.

## Abbreviations

The following abbreviations are used in this manuscript:

ONSs	Oral nutrition supplement(s)
FW	Faltering weight
ASD	Autism spectrum disorder
Codex	Codex Alimentarius
US	United States
EU	European Union
FSDU	Food(s) for special dietary purpose(s)
FSMP	Food(s) for special medical purpose(s)
BMI	Body mass index
NFPE	Nutrition-focused physical exam
AAP	American Academy of Pediatrics
NASPGHAN	North American Society for Pediatric Gastroenterology, Hepatology and Nutrition
GI	Gastrointestinal
PKU	Phenylketonuria
MSUD	Maple Syrup Urine Disease
AI	Artificial intelligence
RCT	Randomized clinical trial
